# A novel prognostic time window based on conditional survival and outcomes analyses of primary liver cancer patients

**DOI:** 10.1002/cam4.4762

**Published:** 2022-04-22

**Authors:** Weicheng Lu, Weifeng Hong, Haibo Qiu, Zhongguo Zhou, Zhonglian He, Weian Zeng, Weiqiang Zhong, Jingdun Xie

**Affiliations:** ^1^ State Key Laboratory of Oncology in Southern China, Department of Anesthesiology Sun Yat‐sen University Cancer Center, Collaborative Innovation for Cancer Medicine Guangzhou China; ^2^ Department of Radiotherapy Zhongshan Hospital, Fudan University Shanghai China; ^3^ State Key Laboratory of Oncology in Southern China, Department of Gastric Surgery Sun Yat‐sen University Cancer Center, Collaborative Innovation for Cancer Medicine Guangzhou China; ^4^ State Key Laboratory of Oncology in Southern China, Department of Hepatobiliary Oncology Sun Yat‐sen University Cancer Center, Collaborative Innovation for Cancer Medicine Guangzhou China; ^5^ State Key Laboratory of Oncology in Southern China, Information Center Sun Yat‐sen University Cancer Center, Collaborative Innovation for Cancer Medicine Guangzhou China

**Keywords:** cirrhosis, conditional survival, liver cancer, logistic regression analysis, recurrence, SEER

## Abstract

**Background:**

Liver cancer is one of the most deadly and prevalent cancers. A routine follow‐up plan for liver cancer is crucial but limited. In the present study, we aimed to disclose possible risk factors and critical survival time windows for primary liver cancer.

**Methods:**

We enrolled 692 liver cancer patients from Sun Yat‐sen University Cancer Center (SYSUCC). Univariate and multivariate logistic regression analyses of cirrhosis and recurrence were conducted. A meta‐analysis was utilized to validate an indication of creatinine (CRE) in recurrence. Conditional survival analysis was performed using the Kaplan–Meier method. The results were further verified by the SYSUCC validation cohort and Surveillance, Epidemiology, and End Results (SEER) validation cohort.

**Results:**

Our results indicated that A/G, history of hepatitis, history of alcohol consumption and platelet (PLT) might be potential prognostic factors for cirrhosis in liver cancer patients. CRE was significantly correlated with recurrence due to various therapies, especially after transarterial embolization. Moreover, 1.5 years to 2 years may be a critical time window for deterioration in survival rate based on the conditional survival analysis.

**Conclusion:**

A/G, history of hepatitis, alcohol consumption and PLT may be potential prognostic factors for cirrhosis in liver cancer patients. More attention should be focused on the renal function when treating the patients due to the significant role of CRE. 1.5 years to 2 years is a critical time window for deterioration in survival rate for liver cancer patients that contributes to determining the optimal follow‐up plan in the future.

## Graphical abstract

A/G, history of hepatitis, history of alcohol consumption and PLT may be potential prognostic factors for cirrhosis in liver cancer patients. CRE is a significant factor in recurrence due to various therapies, especially transarterial embolization, which suggests more attention in renal function. 1.5 years to 2 years is a critical time window for deterioration in survival rate for liver cancer patients, which may contribute to the determination of the optimal follow‐up plan in the future.

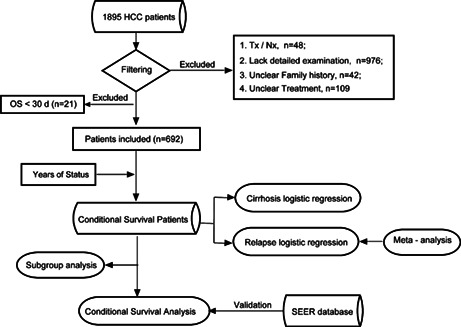

## 1 INTRODUCTION

Primary liver cancer is the sixth most commonly diagnosed cancer and the third leading cause of cancer‐related mortality globally, and it mainly consists of hepatocellular carcinoma (75%–85%) and intrahepatic cholangiocarcinoma (10%–15%). Liver cancer is one of the most deadly and prevalent cancers, with approximately 906,000 new cases and 830,000 deaths in 2020, ranking fifth in global incidence.^1^ Although a growing number of therapies, including surgical resection, chemotherapy, transcatheter arterial chemoembolization, ablation, and immunotherapy, have been approved for the treatment of liver cancer in the past few years,^2^ most have provided limited survival and other outcome benefits,^3^, ^4^ owing to a high incidence of recurrence and metastasis. Moreover, when liver cancer develops in cirrhosis, it may be a leading cause of death, which should be assessed in early diagnosis.^5^

A routine follow‐up plan is vital in liver cancer. It has been suggested that regular follow‐up improves patient prognosis.^6^ Clinically, α‐fetoprotein (AFP) and other potential biomarkers are examined in liver cancer patients every 2 years or more frequently after treatments. Moreover, computed tomography (CT), ultrasonography, or positron emission tomography‐CT is suggested for regular evaluation. However, frequent follow‐up examinations are time consuming and bring certain financial inconveniences. Therefore, it is particularly urgent to formulate a more accurate corresponding follow‐up protocol to evaluate the prognosis of liver cancer patients.

Currently, survival estimates are typically stratified through interrelated risk factors, such as tumor size, lymph nodes, and family history. As in many other malignancies, liver cancer patients have shown higher hazard rates for death in the first few years followed by decreases over time.^7^ Hence, the predictive estimations made at the initial diagnosis are significant. Conditional survival (CS), which has been used to better estimate real‐time prognosis, aims to predict the probability of a patient already surviving for a specific period.^8^ For example, a previously reported regular survival analysis of 2887 liver cancer patients has concluded that the 1‐, 3‐, and 5‐year survival rates are 49.3%, 26.6%, and 19.5%, respectively.^9^ Nevertheless, it has been demonstrated that cancer patients who already survived 3 years have an additional 1‐year CS of 93.7%, and those who already survived 5 years have an additional 1‐year CS of 91%.^10^ Therefore, CS analysis may provide more detailed and meaningful prognostic information for those who survived the previous management.^5^ However, to the best of our knowledge, no investigations have explored early CS and other outcomes, such as early recurrence and metastasis.

**FIGURE 1:**
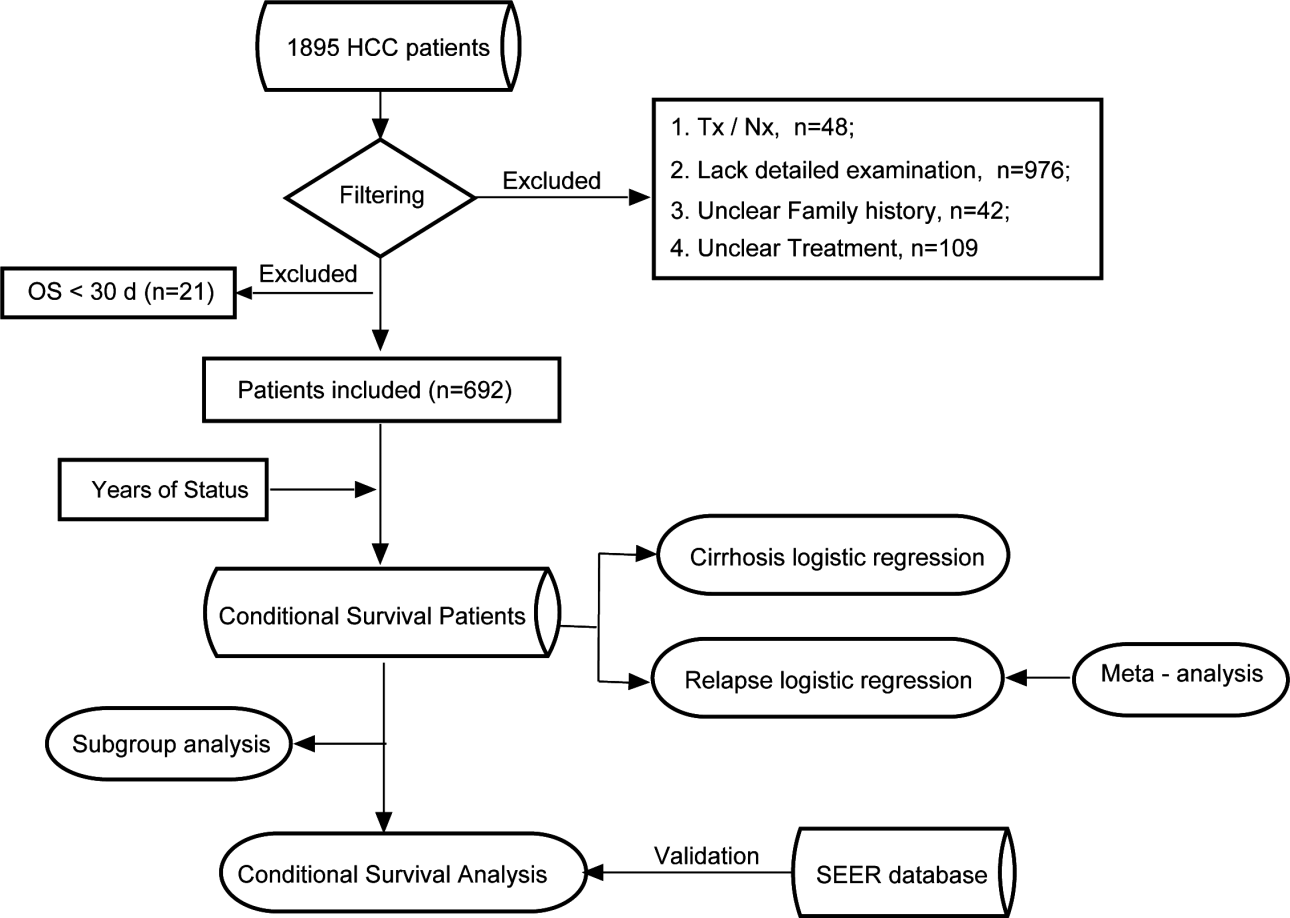
Flow chart of this study

Aiming to describe the short‐term and long‐term survival characteristics, we conducted a retrospective study concerning pathologically diagnosed primary liver cancer patients. Primary liver cancer patients from Sun Yat‐sen University Cancer Center (SYSUCC) were enrolled in our study. Univariate and multivariate logistic regression analyses of cirrhosis and recurrence were conducted. To further validate an indication of creatinine (CRE) in recurrence, we utilized a related meta‐analysis. Conditional survival analysis was performed using the Kaplan–Meier method. The results were verified by the SYSUCC validation cohort and Surveillance, Epidemiology, and End Results (SEER) validation cohort (Figure 1).

## 2 MATERIALS AND METHODS

### 2.1 Patient population

This study was approved by the SYSUCC. We retrospectively identified 1895 liver cancer patients from SYSUCC. Patients were excluded if they had missing data for the clinical and pathological characteristics, including age, sex, family cancer history, TNM stage, survival status, AFP, carcinoembryonic antigen (CEA), or other critical information. A total of 692 patients pathologically diagnosed with liver cancer were included in our population. According to the comparability of the samples, we divided the patients into a validation set and a training set. For further verification, 29,521 liver cancer patients with complete survival status and survival time were selected from the SEER database (www.seer.cancer.gov) as another validation cohort. In addition, we conducted a meta‐study for the logistic regression analysis, including 1964 patients derived from the PubMed database (https://pubmed.ncbi.nlm.nih.gov/) and Embase database (https://www.embase.com/).

### 2.2 Statistical analysis

All statistical analyses were conducted in R software (version 4.0.5) (http://www.r‐project.org/). We analyzed the association between CS and other outcomes, such as cirrhosis, relapse and other clinicopathological characteristics, to identify predictors adjusted for age, first age (age at diagnosis), sex, family cancer history, cirrhosis, treatments, tumor size, lymph node ratio and laboratory test results, including CEA, AFP, platelet (PLT), CRE, albumin–globulin ratio (A/G), glutamyl transpeptidase, glucose, triglyceride (TG), aspartate transaminase (AST), alanine transaminase (ALT), and AST/ALT. Univariate and multivariate logistic analyses were conducted to explore cirrhosis in liver cancer patients. Based on different periods of patients at 3 months, 6 months, 9 months, 1 year, 3 years, 5 years, and 7 years, conditional univariate and multivariate logistic analyses were utilized to assess potential risk factors for recurrence.

### 2.3 Meta‐analysis

#### 2.3.1 Search strategy

We conducted an exhaustive literature search using the following databases without data limitations: PubMed and Embase databases. The literature search date was up to June 20, 2021. The main search terms consisted of “liver cell carcinoma” (also called “hepatocellular carcinoma”, “liver cell carcinoma”, “liver cancer” or “primary liver carcinoma”), and “creatinine” (also called “1‐methylglycocyamidine”, “creatinine hydrochloride”, or “methylglycocyamimine”). The references were also checked for articles that may correlate with our study.

#### 2.3.2 Inclusion and exclusion criteria

The inclusion criteria for the meta‐analysis were as follows: (1) patients with pathologically confirmed primary liver cancer; (2) CRE measured in serum‐based methods; and (3) research demonstrated the correlation between CRE and recurrence as reported by disease‐free survival (DFS) or relapse‐free survival (RFS). The exclusion criteria for the meta‐analysis were as follows: (1) abstracts, letters, reviews, case reports, animal studies, or other nonclinical studies; (2) articles not written in English; (3) studies lacking sufficient information, such as the hazard ratio (HR) and 95% confidence interval (CI); and (4) duplicate data or repeat analysis.

### 2.4 Data extraction, assessment, and statistical analysis

All articles first included were assessed and extracted by two independent authors (W.L. and W.H.). The HR and CI of CRE in RFS or DFS were extracted from the abstracts or full‐text reviews. Disagreement on article selection was discussed until a third author reached a consensus (H.Q.). The following items were recorded for each study: first author, year of publication, country, the total number of cases, gender, follow‐ups, and HRs with 95% CIs. The Newcastle–Ottawa Scale (NOS) was then applied to evaluate the quality of the selected studies.

### 2.5 Conditional survival

Originating from the concept of conditional likelihood in biostatistics by Henson and Ries,^11^ CS is calculated based on conventional Kaplan–Meier or actuarial life table survival data using the following formula: CS(*α*|*β*) = *S*(*α* + *β*)/*S*(*β*). CS predicts the probability of additional *α*‐year survivorship for those who have already survived *β* years after the time of observation. In our study, CS was conducted using the *survival* and *survminer* R packages in the training and validation cohorts, including the samples from the SEER database. To further investigate the time point at which the CS probability dramatically changed, 6 months, 1 year, 2 years, 3 years, and 4 years were selected as additional observational ending times. The crude death rate (CDR) was also calculated to compare deaths over time.

To evaluate the role of clinical characteristics in conditional survival, we classified different subgroups by potentially significant factors. Patients were divided into subgroups according to A/G, AFP, age, ALT, AST, AST/ALT, CEA, CRE, first age, sex, hepatitis, metastasis, PLT, TG, or treatments. Kaplan–Meier analyses were then performed based on the identified subgroups.

## 3 RESULTS

### 3.1 Patient characteristics

Among the 692 patients included, the median age was 60 in the 3‐month group, 6‐month group, 9‐month group, and 1‐year group, while the median age was 61 in the 3‐year group, 63.5 in the 5‐year group, and 66 in the 7‐year group, respectively (Table 1). Among all patients, 23% were younger than the suggested suitable screening age (defined as 45 years old) in the 3‐month group, 6‐month group, 9‐month group, and 1‐year group, and 21%, 20%, and 13% were younger than the suggested suitable screening age in the 3‐year group, 5‐year group, and 7‐year group, respectively. In addition, almost a quarter of patients had a similar family diagnosis history in each group. According to pathological findings, 89% of patients were diagnosed with hepatitis in the 3‐month group, 6‐month group, 9‐month group, 1‐year group, and 3‐year group, while there was a higher rate of patients diagnosed with hepatitis in the 5‐year group (98%) and 7‐year group (100%). Moreover, 39%, 39%, 40%, 40%, 41%, 40%, and 35% of patients had cirrhosis in the 3‐month group, 6‐month group, 9‐month group, 1‐year group, 3‐year group, 5‐year group, and 7‐year group, respectively. The data for the treatments (chemotherapy, surgical resection, embolism, and ablation), other clinical characteristics, examinations, proportion of alcohol consumption history, recurrence, and metastasis are shown in Table 1.

**TABLE 1:** Baseline characteristics

Characteristics	3 months	6 months	9 months	1 year	3 years	5 years	7 years
Age	60 (51–68)	60 (51–68)	60 (51–68)	60 (51–68)	61 (52–68)	63.5 (54–70)	66 (59–71)
First age							
<45	157 (23%)	153 (23%)	146 (23%)	145 (23%)	113 (21%)	33 (20%)	3 (13%)
≥45	535 (77%)	522 (77%)	500 (77%)	496 (77%)	419 (79%)	133 (80%)	20 (87%)
Sex							
Female	93 (13%)	90 (13%)	85 (13%)	85 (13%)	78 (15%)	21 (13%)	5 (22%)
Male	599 (87%)	585 (87%)	561 (87%)	556 (87%)	454 (85%)	145 (87%)	18 (78%)
Hepatitis							
No hepatitis	76 (11%)	74 (11%)	71 (11%)	71 (11%)	59 (11%)	4 (2%)	0 (0%)
Hepatitis	616 (89%)	601 (89%)	575 (89%)	570 (89%)	473 (89%)	162 (98%)	23 (100%)
Cirrhosis							
No	421 (61%)	409 (61%)	388 (60%)	386 (60%)	314 (59%)	99 (60%)	15 (65%)
Yes	271 (39%)	266 (39%)	258 (40%)	255 (40%)	218 (41%)	67 (40%)	8 (35%)
Family history							
No	562 (81%)	548 (81%)	528 (82%)	523 (82%)	429 (81%)	131 (7 s9%)	16 (70%)
Yes	130 (19%)	127 (19%)	118 (18%)	118 (18%)	103 (19%)	35 (21%)	7 (30%)
Therapy							
Chemotherapy	313 (45%)	302 (45%)	280 (44%)	277 (43%)	205 (39%)	68 (41%)	11 (48%)
Resection	212 (31%)	206 (31%)	202 (31%)	202 (32%)	186 (35%)	56 (34%)	8 (35%)
Embolism	40 (6%)	40 (6%)	39 (6%)	39 (6%)	34 (6%)	11 (7%)	2 (9%)
Ablation	124 (18%)	124 (18%)	122 (19%)	120 (19%)	104 (20%)	29 (18%)	2 (9%)
Alcohol history							
No	553 (80%)	539 (80%)	520 (80%)	515 (80%)	428 (80%)	129 (78%)	21 (91%)
Yes	139 (20%)	136 (20%)	126 (20%)	126 (20%)	104 (20%)	37 (22%)	2 (9%)
Metastasis							
No meta	685 (99%)	668 (99%)	639 (99%)	634 (99%)	526 (99%)	165 (99%)	23 (100%)
Meta	7 (1%)	7 (1%)	7 (1%)	7 (1%)	6 (1%)	1 (1%)	0 (0%)
Recurrence							
No recurrence	681 (98%)	664 (98%)	636 (98%)	632 (99%)	523 (98%)	160 (96%)	22 (96%)
Recurrence	11 (2%)	11 (2%)	10 (2%)	9 (1%)	9 (2%)	6 (4%)	1 (4%)
T							
T1‐2	500 (72%)	493 (73%)	484 (75%)	480 (75%)	418 (79%)	145 (87%)	21 (91%)
T3‐4	192 (28%)	182 (27%)	162 (25%)	161 (25%)	114 (21%)	21 (13%)	2 (9%)
N							
N0	672 (97%)	658 (97%)	633 (98%)	628 (98%)	522 (98%)	163 (98%)	22 (96%)
N+	20 (3%)	17 (3%)	13 (2%)	13 (2%)	10 (2%)	3 (2%)	1 (4%)
CEA	2.62 (1.68–3.97)	2.62 (1.69–3.97)	2.59 (1.69–3.92)	2.59 (1.66–3.92)	2.59 (1.65–3.96)	2.59 (1.65–4.05)	2.42 (1.35–4.14)
AFP	67.04 (6.34–714.15)	65.91 (6.37–668.2)	61.47 (6.07–606.85)	59.93 (5.98–609.3)	50.56 (5.59–558.4)	51.92 (6.12–468.2)	28.99 (7.26–616.5)
PLT	140.5 (99–184)	140 (99–183)	140.5 (99.55–183)	141 (100–183)	139 (100–180.25)	141.85 (103.1–177)	122 (101.5–151.5)
CRE	75.2 (64.57–86.12)	75.4 (64.45–86.7)	75.85 (64.73–87.38)	75.8 (64.7–87.3)	75.95 (64.47–87.43)	75.65 (65.73–87.72)	74.02 ± 15.86
A/G	1.35 (1.13–1.51)	1.35 (1.14–1.52)	1.35 (1.16–1.53)	1.35 (1.16–1.52)	1.35 ± 0.28	1.39 ± 0.27	1.32 ± 0.25
GGT	69.7 (43.08–126.08)	69 (42.95–122.65)	67.15 (42.4–116.05)	67.3 (42.7–115)	61.75 (41.05–110.82)	60.75 (38.78–102.35)	45.8 (37–104.2)
GLU	5.28 (4.85–5.92)	5.28 (4.85–5.93)	5.28 (4.85–5.93)	5.28 (4.86–5.92)	5.28 (4.87–5.96)	5.3 (4.84–6.17)	5.46 (4.77–6.76)
TG	0.91 (0.72–1.22)	0.91 (0.72–1.22)	0.92 (0.73–1.23)	0.92 (0.73–1.22)	0.92 (0.74–1.21)	0.92 (0.7–1.19)	0.95 (0.78–1.19)
AST	49.15 (30.08–104.55)	48.7 (30.15–103.45)	48.2 (30–101.95)	48 (30–101.7)	43.95 (29.17–91.7)	48.1 (28.1–101.25)	42.2 (25.15–112.65)
ALT	66.5 (32.32–155.78)	66.6 (32.4–159.35)	66.6 (32.4–161.42)	66.6 (32.4–160.9)	65.25 (32.3–157.1)	81.2 (40.5–181.85)	67.8 (44–169.5)
AST/ALT	0.84 (0.53–1.22)	0.84 (0.53–1.21)	0.82 (0.53–1.2)	0.82 (0.53–1.19)	0.81 (0.5–1.17)	0.7 (0.44–0.97)	0.75 (0.58–0.85)
OS	1303.5 (678.25–1714)	1327 (722.5–1715.5)	1350.5 (799.75–1729.5)	1360 (838–1734)	1467.5 (1137.75–1846.25)	2049 (1882.25–2335.25)	2746 (2616.5–2880.5)
Status							
Alive	683 (99%)	652 (97%)	623 (96%)	591 (92%)	412 (77%)	138 (83%)	19 (83%)
Death	9 (1%)	23 (3%)	23 (4%)	50 (8%)	120 (23%)	28 (17%)	4 (17%)

*Note*: Values are expressed as median (range) in the case of continuous variables and absolute number (percentage) in the case of categorical variables.

Abbreviations: AFP, α‐fetoprotein; A/G, albumin–globulin ratio; ALT, alanine transaminase; AST, aspartate transaminase; CEA, carcinoembryonic antigen; CRE, creatinine; GGT, glutamyl transpeptidase; GLU, glucose; TG, triglyceride; PLT, blood platelet.

### 3.2 Univariate and multivariate logistic regression analyses

**FIGURE 2:**
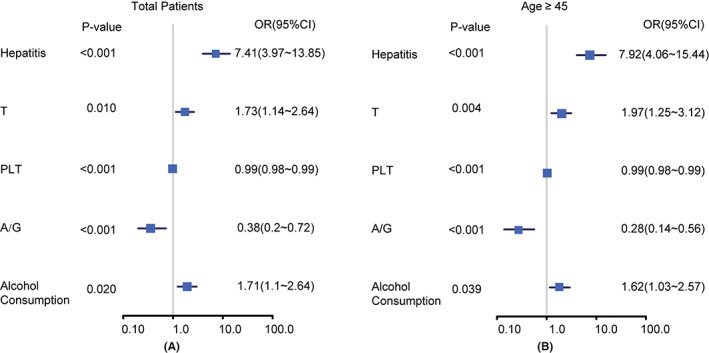
Nomogram for logistic regression results of cirrhosis. Nomogram for logistic regression results about cirrhosis in patients included (A) and older patients (age ≥45), respectively (B)

Univariate analysis of prognostic factors after various treatment options in primary liver cancer was performed. All variables critical in univariate logistic regression analysis (defined as *p* ≤ 0.1) were exposed to multivariate logistic regression analysis. All the regression analysis results of cirrhosis were adapted to construct the nomogram (Figure 2), and the consequences of conditional recurrence are listed in Table 2.

**TABLE 2:** Univariate and multivariate logistic regression models for conditional recurrence at various time points

Characteristics	3 months		6 months		9 months		1 year		3 years		5 years	
	*p*val.*x*	*p*val.*y*	*p*val.*x*	*p*val.*y*	*p*val.*x*	*p*val.*y*	*p*val.*x*	*p*val.*y*	*p*val.*x*	*p*Pval.*y*	*p*val.*x*	*p*val.*y*
A/G	0.920		0.890		0.920		0.960		0.890		0.240	
AFP	0.450		0.470		0.520		0.540		0.550		0.530	
Age	0.120		0.120		0.290		0.270		0.320		0.430	
ALT	0.670		0.670		0.790		0.800		0.800		0.560	
AST	0.940		0.940		0.930		0.990		0.910		0.680	
AST/ALT	0.610		0.600		0.750		0.820		0.710		0.210	
CEA	0.920		0.920		0.930		0.930		0.920		0.590	
Cirrhosis	0.850		0.860		0.700		0.980		0.960		0.630	
CRE	0.070	0.032	0.070	0.032	0.130		0.080		0.080		0.200	
Alcohol history	0.550		0.560		0.040		0.310		0.300		0.510	
Family	0.370		0.360		0.440		0.500		0.460		0.790	
First age	0.260		0.260		0.300		0.350		0.400		0.840	
Sex	0.060		0.050		0.180		0.140		0.190		0.760	
GGT	0.680		0.600		0.360		0.690		0.560		0.270	
GLU	0.650		0.650		0.890		0.730		0.660		0.890	
Hepatitis	0.990		0.990		0.990		0.990		0.990		0.990	
Meta	<0.001	<0.001	<0.001	<0.001	<0.001	<0.001	<0.001	<0.001	<0.001	<0.001	<0.001	
N	0.990		0.990		0.990		0.990		0.990		1.000	
PLT	0.080	0.052	0.080	0.054	0.140		0.310		0.320		0.100	
T	0.990		0.990		0.990		0.990		0.990		0.990	
TG	0.670		0.670		0.870		0.850		0.820		0.080	
Treatments	0.740		0.740		0.740		0.770		0.770		0.940	

*Note*: *P*‐values indicate statistical significance when pval.x <0.1 in the univariate analysis and pval.y <0.05 in the multivariate analysis.

Abbreviations: AFP, α‐fetoprotein; A/G, albumin–globulin ratio; ALT, alanine transaminase; AST, aspartate transaminase; CEA, carcinoembryonic antigen; CRE, creatinine; GGT, glutamyl transpeptidase; GLU, glucose; TG, triglyceride; PLT, blood platelet.

From the nomogram, A/G (*p* = 0.040 for univariate logistic regression; *p* = 0.001 for multivariate logistic regression), history of hepatitis (*p* < 0.001 for univariate logistic regression and multivariate logistic regression), history of alcohol consumption (*p* = 0.040 for univariate logistic regression and *p* = 0.020 for multivariate logistic regression), and PLT (*p* < 0.001 for univariate logistic regression and multivariate logistic regression) were found to be potentially associated with the outcome of liver cirrhosis. Further subgroup analysis suggested that a history of alcohol consumption was more closely associated with cirrhosis in older patients (age ≥45), while it was not significant in younger patients (*p* = 0.3, data not shown). Moreover, A/G, history of hepatitis, and PLT were indicated as possible prognostic factors for cirrhosis after the age of 45. Hence, these findings suggested that there are different potential indicators of prognosis at different ages.

In addition, we found that CRE was significantly correlated with recurrence in primary liver cancer in the first 6 months after treatment (*p* = 0.032 at 0–3 months and 3–6 months), which was also accompanied by metastasis (*p* < 0.001, Table 2).

### 3.3 Meta‐analysis

A meta‐analysis concerning CRE in the recurrence of liver cancer patients was performed to validate our hypothesis. After screening using the NOS, seven studies were included for evaluation. Because only one of the seven studies supported the indication (Table S1), we did not continue to analyze the recurrence using a standard method.

### 3.4 Conditional survival

**FIGURE 3:**
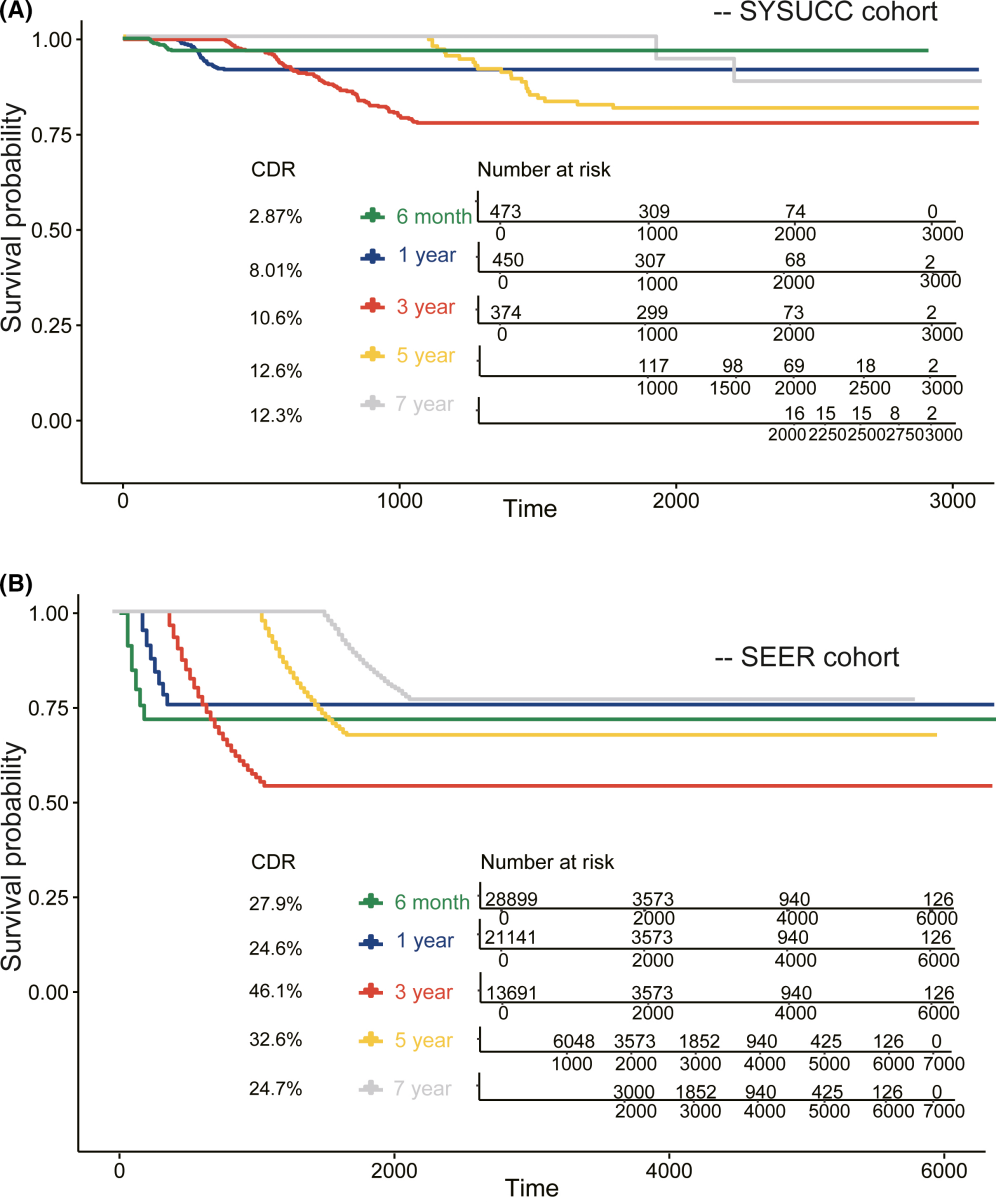
The conditional probability of survival curves at various time points from liver cancer patients. Traditional Kaplan–Meier method estimates of survival rates in 3 months−6 months (green line), 6 months–1 year (blue line), 1 year–3 years (red line), 3 years–5 years (yellow line), 5 years–7 years (gray line) from SYSUCC training cohort (A) and SEER validation cohort (B). SEER, Surveillance, Epidemiology, and End Results; SYSUCC, Sun Yat‐sen University Cancer Center

We assessed the CS statuses at 0–1 year, 1–3 years, 3–5 years, and 5–7 years. A total of 692 patients were divided into a training cohort and a validation cohort. A significant decline was observed in the survival curve at 1–3 years, and this trend was partially rescued at 3–7 years, indicating that liver cancer patient survival may deteriorate within 1–3 years (Figure 3A). The CDR was also significantly increased from 8.01% to 10.6%, and this finding was further confirmed through the different CS curves of the patients in the validation set (Figure S2) and the SEER cohort (Figure 3B).

**FIGURE 4:**
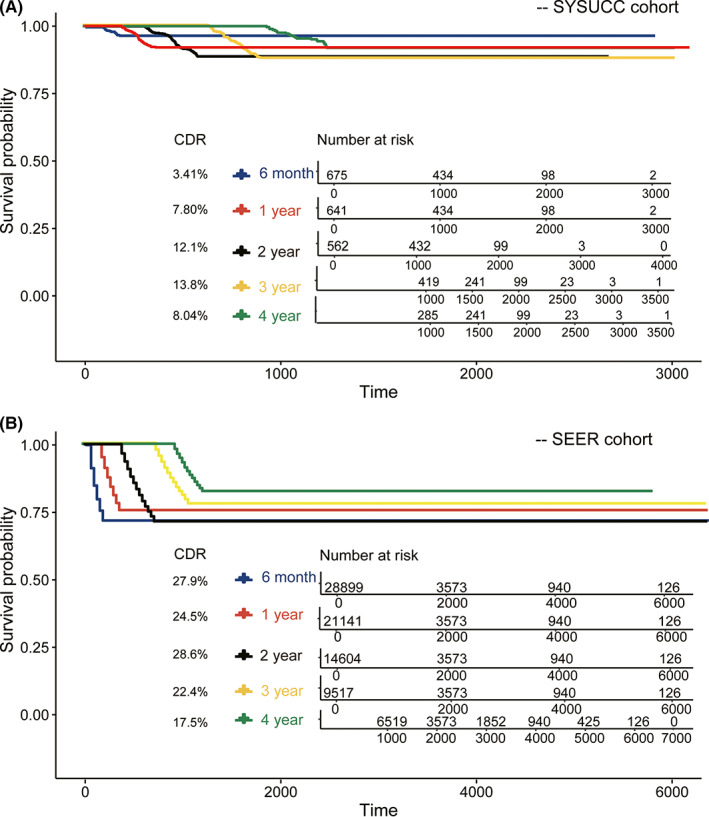
The conditional probability of survival curves at more detailed time points from liver cancer patients. Traditional Kaplan–Meier method estimates of survival rates in 3 months–6 months (blue line), 6 months–1 year (red line), 1 year–2 years (black line), 2 years– 3 years (yellow line), 3 years–4 years (green line) from total SYSUCC cohort (A) and SEER cohort (B). SEER, Surveillance, Epidemiology, and End Results; SYSUCC, Sun Yat‐sen University Cancer Center

To identify a more accurate time window for potential deterioration of liver cancer, we subdivided the different survival times of all patients (0–6 months, 6 months−1 year, 1–2 years, 2–3 years, and 3–4 years) and then conducted a CS analysis at different time points. The results indicated that the survival probability significantly decreased between 1.5 and 2 years after treatment according to the survival curve (Figure 4A) as the CDR increased from 7.80% to 12.1%. Hence, we hypothesized that 1.5–2 years may be a crucial time window for the maintenance of liver cancer. The SEER cohort further validated this hypothesis (Figure 4B).

To elucidate potential factors involved in the 1.5–2 years deterioration time window, we conducted subgroup survival analysis with different clinical characteristics (Table 3). According to the results, A/G, AFP, AST/ALT, and treatments may play a key role in the deterioration time window. Moreover, we demonstrated that the proportion of patients who had hepatitis gradually increased in both the dead and alive groups in the CS of 1–4 years (Figure S3A). Among the early stages of CS, patients who were treated with chemotherapy accounted for a relatively larger proportion (Figure S3B). There were no significant differences between patients in this time window in terms of the history of alcohol consumption and age at first diagnosis (Figure S3C,D).

**TABLE 3:** Subgroup survival analysis with different clinical characteristics

	6 months	1 year	2 years	3 years	4 years
A/G	0.02**9**	0.046	<0.001	0.044	0.180
AFP	0.061	<0.001	0.027	0.055	0.460
Age	0.083	0.097	0.068	0.760	0.580
ALT	0.770	0.100	0.017	0.320	0.420
AST	0.260	0.0092	0.190	0.110	0.470
AST/ALT	<0.001	0.027	<0.001	0.400	0.042
CEA	0.710	0.320	1.000	0.610	0.200
CRE	0.720	0.280	0.450	0.170	0.690
First age	0.900	0.380	0.860	0.190	0.680
Sex	0.560	0.042	0.950	0.170	0.870
Hepatitis	0.740	0.880	0.038	0.940	0.250
Alcohol history	0.021	0.054	0.770	0.031	0.240
Meta	0.600	0.410	0.330	<0.001	<0.001
PLT	0.470	0.950	0.390	0.380	0.610
TG	0.670	0.320	0.93	0.820	0.400
Treatments	0.036	<0.001	<0.001	<0.001	<0.001

*Note*: Analyses indicate statistical significance when *p* < 0.05.

Abbreviations: AFP, α‐fetoprotein; A/G, albumin–globulin ratio; ALT, alanine transaminase; AST, aspartate transaminase; CEA, carcinoembryonic antigen; CRE, creatinine; GGT, glutamyl transpeptidase; GLU, glucose; TG, triglyceride; PLT, blood platelet.

**FIGURE 5:**
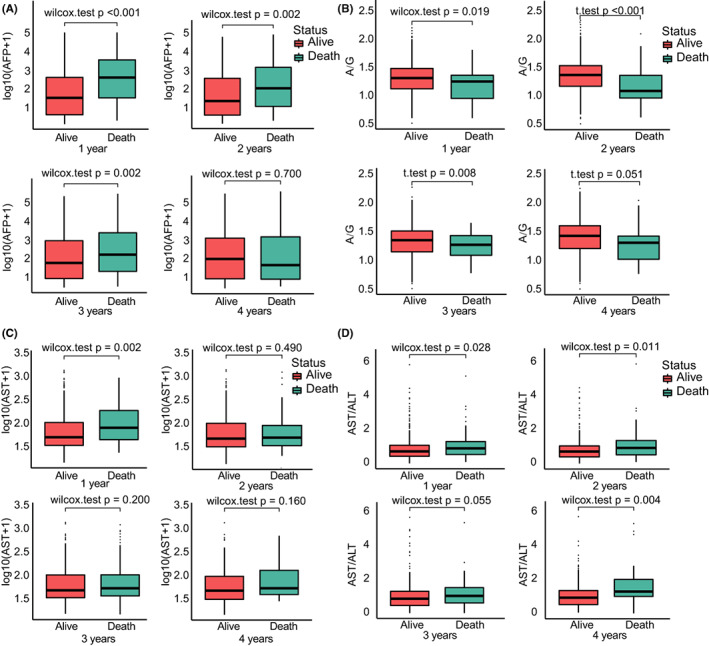
AFP, A/G, AST, AST/ALT in groups with different survival statuses from 1 year to 4 years. AFP (A), A/G (B), AST (C), AST/ALT (D) show the different trends in liver cancer patients in 1 year, 2 years, 3 years, 4 years. AFP, α‐fetoprotein; A/G, albumin–globulin ratio; ALT, alanine transaminase; AST, aspartate transaminase

We further investigated the detailed difference between groups in different statuses. AFP showed a significant difference among various statuses in early stages, including 0–1 year (*p* < 0.001), 1–2 years (*p* = 0.002), and 2–3 years (*p* = 0.002). A/G demonstrated an opposite trend at 0–1 year (*p* = 0.019), 1–2 years (*p* < 0.001), and 2–3 years (*p* = 0.008). However, there was no noticeable difference for single ALT in each stage (data not shown), while single AST was predominantly upregulated at 0–1 year (*p* = 0.002). Nevertheless, the DeRitis ratio, which is defined as the ratio of AST to ALT, was significantly upregulated in the 0–1 year (*p* = 0.028) and 1–2 year stages (*p* = 0.011) (Figure 5). These results indicated the potential correlation between liver damage and survival prognosis.

## 4 DISCUSSION

Primary liver cancer is an increasingly prevalent disease globally, ranking as the third leading cause of cancer‐related deaths worldwide. Although great progress has been made in liver cancer therapy, the average 5‐year survival rate is only 5%–9% due to metastasis, recurrence, and diverse complications in maintaining the neoplasm. Although many studies have investigated the outcomes of liver cancer patients after various treatments, the prognostic survival of liver cancer patients remains poor. Hence, there is an urgent need to discover prognostic factors that contribute to the plan for follow‐up.

Herein, we demonstrated that A/G level, history of hepatitis, history of alcohol consumption, and PLT level were significantly associated with liver cirrhosis in liver cancer patients, which was consistent with the phenomenon in the clinic. Our results also implied that clinical examinations show different sensitivities depending on patient age. For example, more attention should be given to whether elderly patients (age ≥45) have cirrhosis at the same time as liver cancer. PLT and other liver function factors should be further evaluated to guide treatment options for younger patients.

We also illustrated the potential relationship between CRE and liver cancer recurrence. Numerous studies have explored the underlying mechanism but failed to reach a consistent conclusion. Faillaci et al. demonstrated that CRE does not correlate with relapse in hepatocellular cancer patients after direct‐acting antiviral treatment.^12^ At the same time, Yang et al. found that CRE is related to a higher risk of relapse after ablation of hepatocellular carcinoma.^13^ It remains unknown whether CRE could be a potential prognostic factor for liver cancer. Based on our logistic analysis and meta‐analysis, we hypothesized that CRE plays a role in the recurrence of liver cancer. Although the conclusions were not consistent, we speculated that the relevance of CRE mainly depends on patients' treatment options, especially in transarterial embolization treatment, which may causes renal damage. Our study adds to the accumulating evidence that an increase in CRE indicates renal dysfunction, thus indirectly resulting in recurrence and other poor outcomes. Therefore, when selecting treatment options for patients, attention should be focused on the potential renal damage caused by the therapeutic schedule.

Because conventional actuarial OS estimates rely exclusively on static factors determined around the time of surgery, CS analysis sheds new light on a better assessment of survival time at each follow‐up. CS analysis has been widely applied in the analysis of renal cell carcinoma,^14^ colorectal cancer,^15^ diffuse large B‐cell lymphoma,^16^ upper tract urothelial carcinoma,^17^ and liver cancer.^5^, ^7^ For patients treated with hepatic arterial infusion chemotherapy with or without concurrent radiotherapy, Cho et al. found that those who had already survived for 0, 1, 2, and 3 years, the CS estimates of surviving an additional 2 years are 35.6%, 55.1%, 82.0%, and 77.4%, respectively.^18^ For intrahepatic cholangiocarcinoma patients, OS decreases over time from 39% at 3 years to 16% at 8 years, while 3‐year CS increases over time among those patients who survived.^19^ Conditional DFS of patients who underwent resection of neuroendocrine liver metastasis also exponentially improved as patients survived additional years without recurrence, thereby providing more accurate prognostic measures compared to traditional DFS measures.^20^ Other studies have also illustrated that CS provides more accurate prognostic information for hepatocellular carcinoma patients and physicians after colorectal liver metastasis resection,^21^ hepatic resection^22^ or liver transplantation.^23^ A previous result from the SEER database has indicated that conditional overall and cause‐specific survival improve in the positive/elevated AFP group.^24^ In conclusion, CS is a more relevant measure of prognosis in surviving patients over time. Because CS provides more critical quantitative information, it may be of significant value to patients and health care professionals.

According to more than 10 years of experience at the University of Bologna,^25^ increased survival is most likely the result of more stringent follow‐up, increased accuracy in detecting recurrence at earlier stages, and consequently more opportunities for a potential cure when treating recurrent tumors. However, there are few studies on actual CS to guide prognosis for liver cancer within 1–2 years. Accurate follow‐up time windows are needed. In our study, we evaluated CS based on different early times. Surprisingly, we found a significant decline in the survival curve of 1–3 years among the cancer patients. Further validation identified 1.5–2 years as a critical time window for deterioration in the survival rate, implying that patients should pay more attention to the changes in their conditions during this period, thus adjusting their therapies and follow‐up plans in time. The present study also demonstrated that decreased serum levels of A/G, increased AFP, and AST/ALT were associated with poor survival. In addition, the history of hepatitis implied a worse outcome in our cohort. To better understand the underlying correlation between conditional and prognostic examinations, we utilized differential abundance analysis between groups in different statuses from 1 year to 4 years. We observed that the conditional and prognostic examinations played various roles in different periods, and A/G, AFP, and AST/ALT were indicated to be significant.

The present study had several limitations. First, the present study was susceptible to biases because all the analyses were conducted based on retrospective data. Moreover, only a small number of pathologically diagnosed samples were included in the present study, and metastasis was not investigated due to the limited number of patients. In addition, other potential factors, such as comorbidities, were not included. The relationship between endogenous CRE clearance and recurrence of liver cancer was not evaluated due to the lack of information. In addition, we did not further investigate the patients regarding treatment modalities, which will be explored in the future.

Despite these limitations, our results indicated that A/G level, history of hepatitis, alcohol consumption, and PLT are potentially associated with liver cirrhosis when considering age. Moreover, CRE may be significantly correlated with the outcome of recurrence in primary liver cancer in the first 6 months after treatment, especially after transarterial embolization. Our results also suggested a potentially critical time window for deterioration in survival rate based on the CS analysis, which may contribute to the determination of the optimal follow‐up plan in the future.

## CONFLICT OF INTEREST

None of the authors has personal or financial conflict of interest to declare in relation to this publication.

## AUTHOR CONTRIBUTIONS

Jingdun Xie and Weiqiang Zhong conceived and designed most of the project. Zhonglian He, Zhongguo Zhou, and Weian Zeng collected clinic samples, Weicheng Lu, Weifeng Hong, and Haibo Qiu conducted experiments and analyzed the data. Weicheng Lu and Weifeng Hong organized the figures and tables. All authors wrote the manuscript together and approved the final manuscript.

## ETHICAL APPROVAL STATEMENT

This study was approved by the ethics committee of Sun Yat‐sen University Cancer Center (RDDA2021594509).

## Supporting information


Figure S1

Figure S2

Figure S3

Table S1

## Data Availability

The data that support the findings of this study are available on request from the corresponding author. The data are not publicly available due to privacy or ethical restriction.
