# Supplementary figures and images for: A novel prognostic time window based on conditional survival and outcomes analyses of primary liver cancer patients
Source: Cancer Med. 2022 Apr 22;11(20):3873–85. doi: 10.1002/cam4.4762 (PMC9582677; doi:10.1002/cam4.4762)

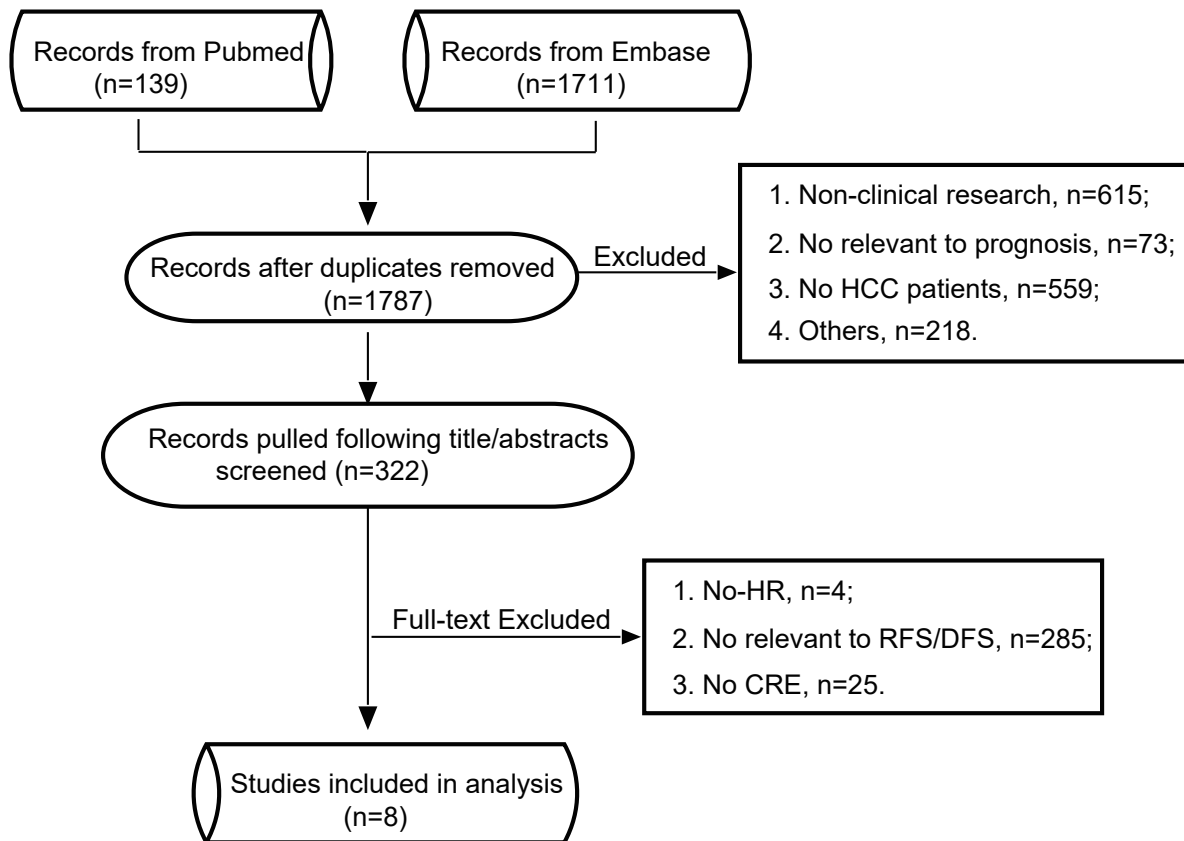

Supplement: Supplementary file 1 — Figure S1 [file CAM4-11-3873-s004.pdf]

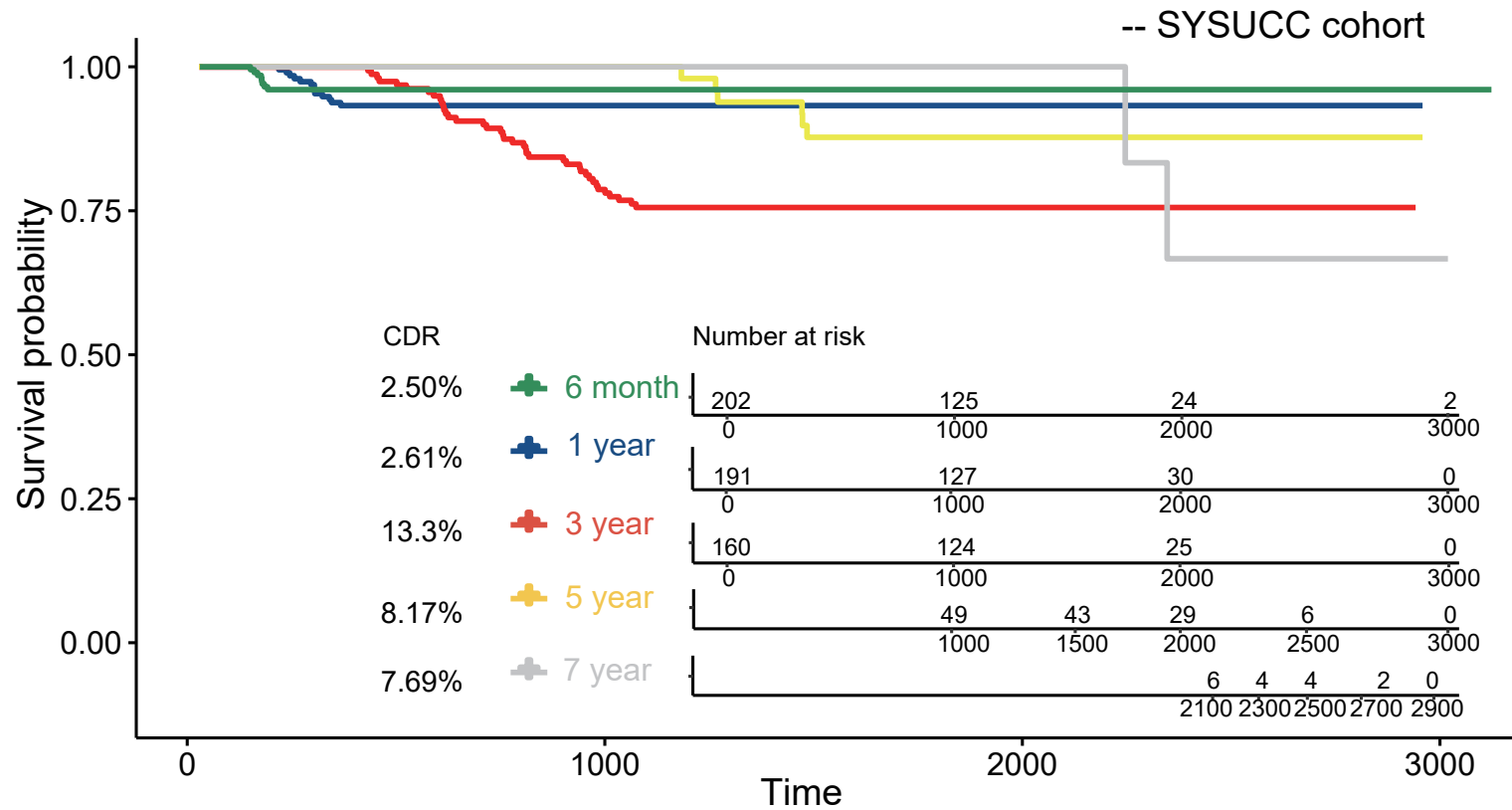

Supplement: Supplementary file 2 — Figure S2 [file CAM4-11-3873-s002.pdf]

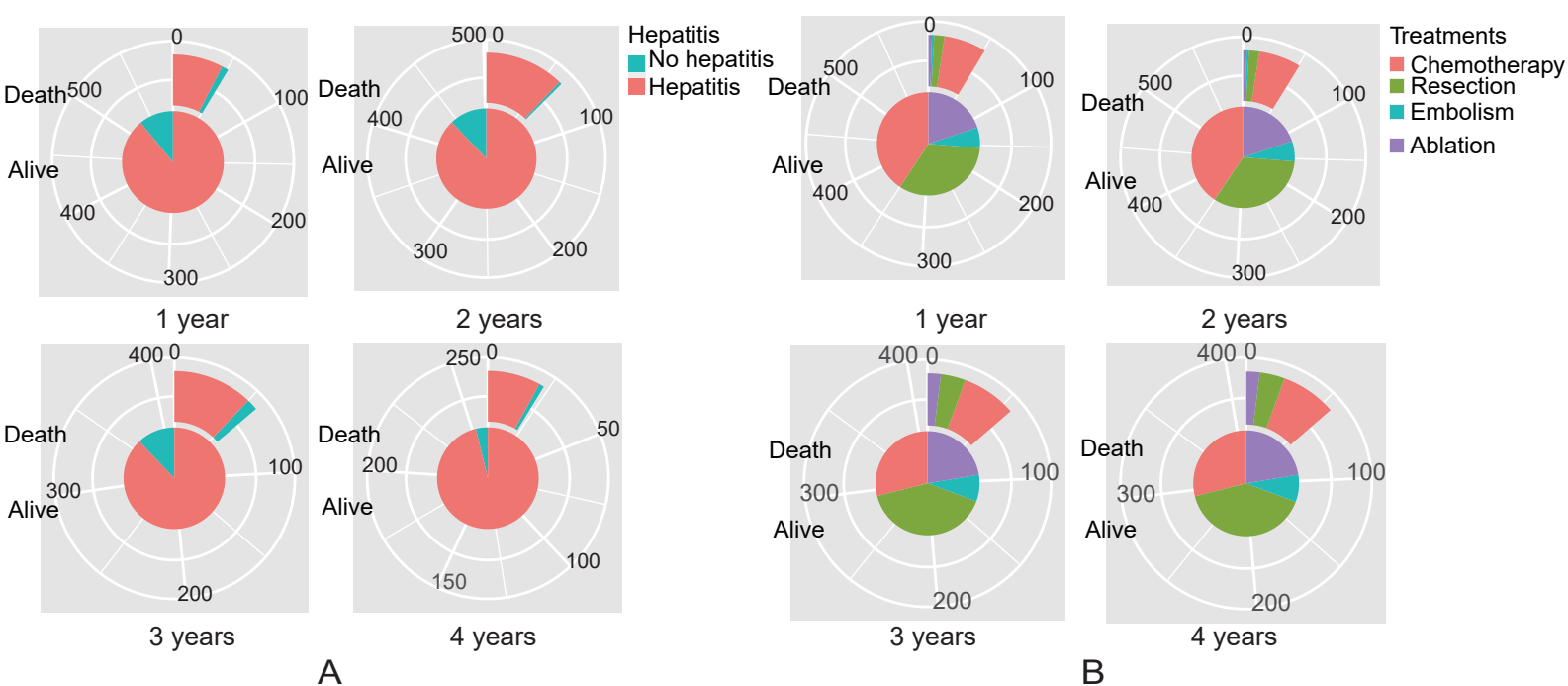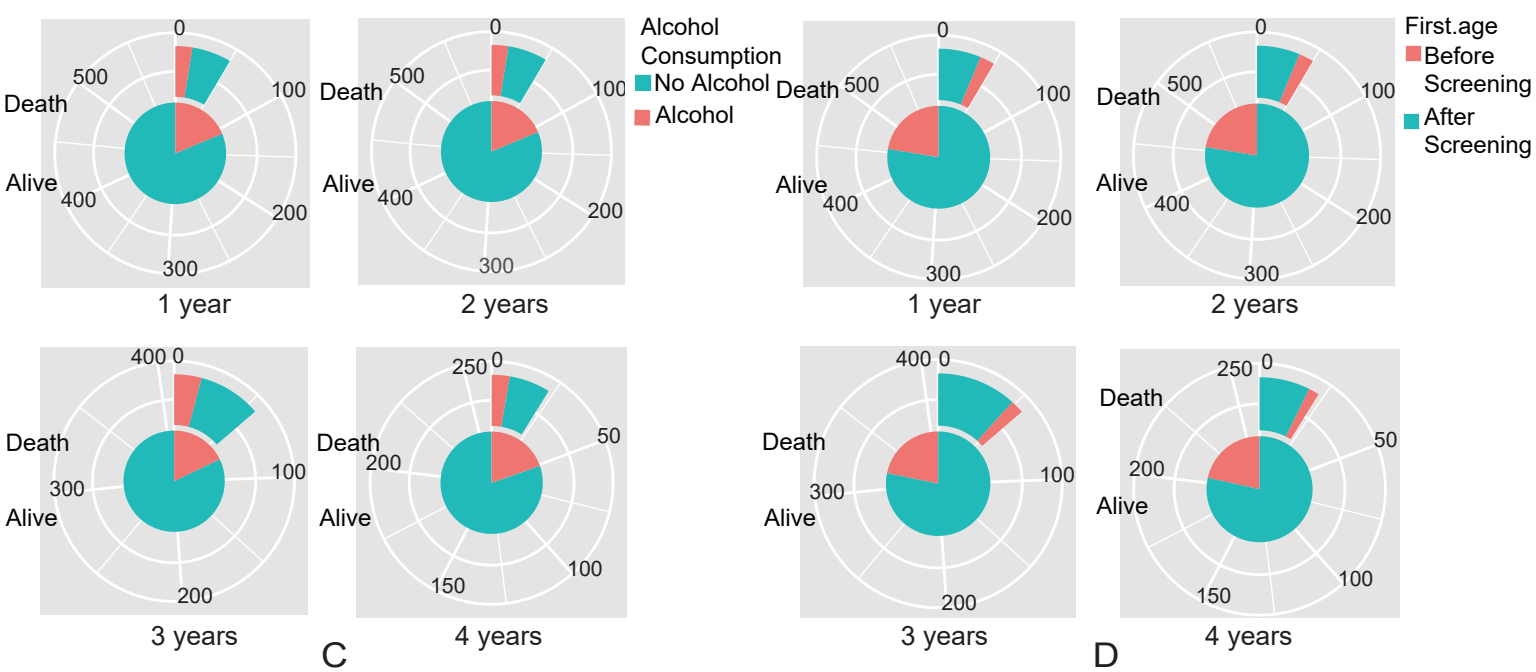

Supplement: Supplementary file 3 — Figure S3 [file CAM4-11-3873-s003.pdf]
