# Supplementary material for: A novel prognostic time window based on conditional survival and outcomes analyses of primary liver cancer patients
Source: Cancer Med. 2022 Apr 22;11(20):3873–85. doi: 10.1002/cam4.4762 (PMC9582677; doi:10.1002/cam4.4762)
Supplement: Supplementary file 4 — Table S1 [file CAM4-11-3873-s001.docx]

**Supplemental table1. Main characteristics of all the studies included in the meta-analysis**

| **Author** | **Year** | **Journal** | **Male/Female** | **HR/OR** | **LL** | **UL** | **P.value** |
| --- | --- | --- | --- | --- | --- | --- | --- |
| XP Yan.et | 2016 | Medicine | 47（38/9） | 1.015 | 0.993 | 1.037 | 0.195 |
| Chuang-Yu Chang. et | 2020 | Journal of Chinese Medical Association | 420（278/142） | 1.073 | 0.820 | 1.403 | 0.608 |
| Hongcai Yang.et | 2020 | Cancer Biol Med | 921（754/167） | 1.002 | 1.000 | 1.004 | 0.012 |
| Darren W.Chua.et | 2018 | European Journal of durgical oncology | 49（42/7） | 0.983 | 0.998 | 1.171 | 0.089 |
| Dong hui Lu.et | 2018 | Oncology letter | 369（248/121） | 1.002 | 0.987 | 1.017 | 0.819 |
| Yiquan Jiang.et | 2018 | Journal of cancer | 213（106/17） | 1.000 | 1.000 | 1.020 | 0.290 |
| Francesca Faillaci.et | 2018 | Hepatology | 35(30/5) | 1.310 | 0.217 | 7.894 | 0.768 |

HR: hazard ratio; OR, odds ratio; LL, lower confidence intervals; UL, upper confidence intervals. All the values were obtained by reporting in text, respectively.
